# Nociceptive Sensory Neurons Mediate Inflammation Induced by *Bacillus Anthracis* Edema Toxin

**DOI:** 10.3389/fimmu.2021.642373

**Published:** 2021-08-03

**Authors:** Nicole J. Yang, Dylan V. Neel, Liwen Deng, Michelle Heyang, Angela Kennedy-Curran, Victoria S. Tong, Jin Mo Park, Isaac M. Chiu

**Affiliations:** ^1^Department of Immunology, Harvard Medical School, Boston, MA, United States; ^2^Cutaneous Biology Research Center, Massachusetts General Hospital and Harvard Medical School, Charlestown, MA, United States

**Keywords:** *Bacillus anthracis*, edema toxin, nociceptors, neurogenic inflammation, neuron

## Abstract

Bacterial products are able to act on nociceptive neurons during pathogenic infection. Neurogenic inflammation is an active part of pain signaling and has recently been shown to impact host-pathogen defense. *Bacillus anthracis* Edema Toxin (ET) produces striking edema in peripheral tissues, but the cellular mechanisms involved in tissue swelling are not completely understood. Here, we find that nociceptive neurons play a role in ET-induced edema and inflammation in mice. Subcutaneous footpad infection of *B. anthracis* Sterne caused ET-dependent local mechanical allodynia, paw swelling and body weight gain. Subcutaneous administration of ET induced paw swelling and vascular leakage, the early phases of which were attenuated in the absence of Trp_v_1^+^ or Na_v_1.8^+^ nociceptive neurons. Nociceptive neurons express the anthrax toxin receptor ANTXR2, but this did not mediate ET-induced edema. ET induced local cytokine expression and neutrophil recruitment, which were dependent in part on Trp_v_1^+^ nociceptive neurons. Ablation of Trp_v_1^+^ or Na_v_1.8^+^ nociceptive neurons also attenuated early increases in paw swelling and body weight gain during live *B. anthracis* infection. Our findings indicate that nociceptive neurons play an active role in inflammation caused by *B. anthracis* and Edema Toxin to potentially influence bacterial pathogenesis.

## Introduction

Nociceptive sensory neurons densely innervate barrier tissues such as the skin, lung and gut, and detect physical and chemical stimuli that are potentially damaging, which initiates pain signaling. Recent work has shown that nociceptive neurons can also detect bacterial products and respond by secreting neuropeptides and initiating pain behavior (1–3). Nociceptor activation triggers neurogenic inflammation, an axonal reflex that leads to rapid release of neural mediators from peripheral nerve terminals that in turn act on the vasculature and immune system to drive tissue inflammation (4). Major nociceptive neuron mediators include the neuropeptides calcitonin gene-related peptide (CGRP) and substance P (SP). CGRP acts on vascular smooth muscle cells to promote vasodilation and SP acts on vascular endothelial cells to increase vascular permeability, leading to edema (4). However, the role of the nervous system in mediating tissue inflammation in response to pathogen exposure is only beginning to be understood. Recent work demonstrated that during bacterial infection, neuropeptides can additionally act on innate immune or epithelial cells to influence antimicrobial immunity (3, 5, 6). These findings suggest that nociceptive neurons may play a broader role in modulating host defense against pathogenic insults.

*Bacillus anthracis* is a gram-positive, spore-forming bacterium which is the etiologic agent of anthrax. Infection is classified based on the entry route of spores, and can produce cutaneous, inhalational and gastrointestinal disease. Cutaneous anthrax is characterized by extensive soft tissue edema which may extend beyond the local site of infection, and the formation of a black, painless eschar (7). Fluid accumulation in the chest or abdomen are also typical of inhalational and gastrointestinal anthrax (7), making edema a key feature of *B. anthracis* infection. However, the cellular and molecular mechanisms leading to local and systemic edema are not well understood.

Anthrax toxins are the key virulence factors of *B. anthracis*, and consist of Protective antigen (PA), Edema Factor (EF) and Lethal Factor (LF). PA binds to its cognate receptors ANTXR1 and ANTXR2, with higher affinity to the latter, and oligomerizes into a pore that translocates EF and LF into the cytoplasm. EF is an adenylyl cyclase that generates cAMP and LF is a protease that cleaves MAPK kinase kinases (MAPKKs), and they collectively produce potent alterations in host intracellular signaling. We recently reported that nociceptive neurons express ANTXR2 and can be targeted by anthrax toxins to alter cAMP levels and alter pain behavior (8). This finding suggested that these neurons may potentially be involved in pathogenesis during *B. anthracis* infection.

Administration of Edema Toxin (ET), the combination of PA and EF, produces striking edema and vascular leakage in laboratory animals (9, 10). Previous studies have suggested direct and indirect actions of ET on the vasculature to mediate tissue swelling. EF reduces cadherin expression and weakens intercellular junctions in endothelial cell lines, potentially contributing to the vascular effusion observed *in vivo* (11). In addition, chemical inhibition of prostanoid, neurokinin and histamine pathways attenuated ET-induced edema in rabbits, suggesting a potential role for mast cells and sensory neurons (12). Overall, the molecular and cellular interactions induced by ET remain incompletely defined.

Here, we report that nociceptive neurons contribute to ET-induced edema and inflammation. In a subcutaneous footpad infection model of *B. anthracis* Sterne in mice, we found that bacterial infection produces significant swelling accompanied by mechanical allodynia, both of which are dependent on EF, the effector component of ET. Nociceptor ablation by chemical or genetic methods significantly attenuated early tissue swelling, vascular leakage, and neutrophil recruitment induced by ET. Nociceptors also contributed to the early phase of *B. anthracis* induced edema. Edema was independent of ANTXR2 on nociceptive neurons, suggesting that nociceptors respond indirectly to infection and modulate tissue edema. Our findings highlight a novel regulatory role for nociceptive neurons in driving edema and inflammation during *B. anthracis* infection.

## Materials And Methods

### Animals

All animal experiments were approved by the Institutional Animal Care and Use Committee (IACUC) at Harvard Medical School. C57BL/6J mice were purchased from Jackson Laboratory (Bar Harbor, ME) and bred at Harvard Medical School. All mice were housed in individually ventilated microisolator cages within a full barrier, specific pathogen-free animal facility at Harvard Medical School. Mice were kept on a 12 hr light/dark cycle and provided *ad libitum* access to food and water. Na_v_1.8-lineage neuron-depleted mice (Na_v_1.8^cre/+^/DTA^+/-^) and control littermates (Na_v_1.8^+/+^/DTA^+/-^) were generated by breeding Na_v_1.8-cre knock-in mice (provided by J. Wood, University College London) with B6.Rosa26-stop(flox)-DTA mice (Jackson Laboratory) as previously described (6). Na_v_1.8 neuron-specific conditional ANTXR2 mice (Na_v_1.8^cre/+^/Antxr2^fl/fl^) were generated by breeding Nav1.8-cre knock-in mice with a conditionally targeted allele of Antxr2 in the transmembrane region (Antxr2^fl/fl^, Jackson Laboratory) as previously described (8). Experiments were performed with age- and sex-matched mice between 7 to 14 weeks of age unless otherwise noted.

### Drug Treatment

For chemical ablation of Trp_v_1^+^ neurons with systemically administered resiniferatoxin (RTX, Sigma Aldrich), 4-week-old C57BL/6 mice were injected subcutaneously in the flank with escalating doses of RTX (30, 70, 100 µg/kg on consecutive days) or vehicle (2% DMSO/0.15% Tween-80 in PBS). For chemical ablation of Trp_v_1^+^ neurons with intrathecally administered RTX, 4-week-old C57BL/6 mice were injected intrathecally near the iliac crest with two daily doses of RTX (25 ng) or vehicle (0.25% DMSO/0.02% Tween-80 in PBS). BIBN 4096 (Tocris; 50 pmol in 10 µL) and its vehicle (0.05% DMSO in saline), Spantide I (Tocris; 5 nmol in 10 µL) and its vehicle (water) were injected subcutaneously into the ipsilateral footpad using a 100 µL Hamilton syringe and 32-gauge needle under isoflurane anesthesia. The antagonists and vehicle controls were administered 15 min prior to ET.

### Recombinant Anthrax Toxins

Protective antigen (PA) was obtained through BEI Resources (#NR-140, recombinant from *B. anthracis*). The Edema Factor (EF) clone used in this study contains an extra alanine at the N-terminus compared to the native sequence (**Supplementary Figure 5**), which enhances intracellular stability and activity (13). EF was expressed in Rosetta 2 (DE3) *E. coli* (Novagen) using the Champion pET SUMO expression system (ThermoFisher Scientific) and purified as previously described (8). Endotoxin levels in the final product measured using the Pierce LAL Chromogenic Endotoxin Quantitation Kit (ThermoFisher Scientific) was 0.93 EU/mg.

### *B. anthracis* Culture and Infection

The *Bacillus anthracis* Sterne 7702 strain (BA663, #NR-9396) and its isogenic mutant lacking EF (BA695, #NR-9398) were obtained from ATCC. Spores for both strains were prepared as previously described (14) and stored at 4°C. All procedures were approved by the Committee on Microbiological Safety (COMS) at Harvard Medical School and conducted under Biosafety Level 2 protocols and guidelines. For infection, *B. anthracis* spores were streaked on BHI agar plates and grown overnight at 37°C. A day prior to infection, bacterial colonies were picked and inoculated into 2x SG medium and grown overnight at 37°C, 250 rpm. The overnight culture was diluted 50-fold into fresh 2x SG medium and grown for approximately 2 hr until mid-log phase was reached, then washed twice with PBS and stored in PBS on ice until injection. Mice were anesthetized with 3% isoflurane (Patterson Veterinary) with oxygen using a precision vaporizer, after which the indicated dose of bacteria was injected subcutaneously into the left hind paw in a 20 µL volume using a 100 µL Hamilton syringe and 31-gauge needle. The inoculum was also plated on BHI agar plates and incubated overnight to confirm dosages. Survival, paw thickness and body weight were monitored daily post-infection.

### Tissue Bacterial Load Measurements

Mice were infected *via* subcutaneous footpad injection of vegetative *Bacillus anthracis* Sterne 7702 at a dosage of 1 × 10^7^ CFUs, following procedures described in *B. anthracis* culture and infection. At 5 or 48 hr post-infection, mice were euthanized by CO_2_ asphyxiation and the ipsilateral foot was harvested below the ankle. The ipsilateral popliteal lymph node, liver and spleen were also harvested. The organs were immediately transferred into pre-weighed 2 mL microcentrifuge tubes containing 1 mL of cold PBS and stored on ice. After organ weights were measured, foot samples were minced with scissors to facilitate homogenization. All samples were homogenized by bead beating at 25 Hz for 10 min using a TissueLyser (Qiagen). The tissue lysate was serially diluted in PBS and plated on BACARA agar (Biomerieux), a chromogenic media selective for the *Bacillus cereus* group (15). Plates were incubated overnight at 37°C and the number of resulting colonies were counted to calculate bacterial loads. Total CFU counts were normalized to organ weight.

### Intraplantar Injection of Edema Toxin

2 µg of PA and 2 µg of EF were mixed with PBS to a volume of 20 µL. Injection was performed with a 100 µL Hamilton syringe and 32-gauge needle under isoflurane anesthesia.

### Paw Thickness Measurements

Paw thicknesses were measured under isoflurane anesthesia using digital calipers (VWR International) and normalized to initial values measured prior to injection of bacteria or toxin.

### Measurement of Mechanical Sensitivity

All behavioral tests were performed by observers blinded to the treatment groups or genotypes. Treatment groups were randomized and evenly distributed across cages and sex. Mechanical sensitivity thresholds were measured using von Frey filaments and the up/down method as previously described (8). Briefly, mice were habituated on the behavior apparatus for 2 consecutive days for 1 hr each. After habituation, baseline measurements were obtained on 3 separate days and averaged prior to infection. After infection, measurements were made at the indicated timepoints up to 48 hr post-infection.

### Hot Plate Test

Mice were placed on a temperature-controlled metal plate (IITC Life Science) at 55°C, and the latency to response (jumping or hind paw licking) was recorded. A 90 s cut-off was imposed to avoid tissue injury.

### Fluid Content Measurements

C57BL/6 mice were given subcutaneous footpad injection of 1×10^7^ CFU *Bacillus anthracis* Sterne as described in *B. anthracis* culture and infection. At 48 hr post-infection, the indicated organs were harvested and weighed to measure their wet weight. Following incubation at 70°C for 40 min and 55°C for 48 hr with open lids, dry weights were recorded. Fluid weight was calculated as wet weight – dry weight, then normalized to dry weight.

### Evans Blue Extravasation (Miles Assay)

Evans Blue dye (Sigma Aldrich) dissolved in 0.9% saline was administered retro-orbitally (25 mg/kg) and allowed to circulate for 30 min, after which mice were given intraplantar injection of ET. At 5 hr post-injection, animals were put under terminal anesthesia with Avertin (500 mg/kg, i.p) and perfused with 10 mL of cold PBS. The glabrous skin of the ipsilateral footpad was collected using a razor blade and minced with scissors in 500 µL of 50% TCA in 0.9% saline. The tissue was further homogenized in a TissueLyser II (Qiagen) for 10 min at 30 Hz, and centrifuged at 18,000 xg for 10 min. 100 µL of the supernatant was transferred to a 96-well plate and the absorbances at 620 nm were measured using a Synergy Mx multi-mode microplate reader (BioTek), along with a standard curve of Evans Blue prepared in 50% TCA. Values were fitted using GraphPad Prism.

### Neutrophil Influx

Mice were given intraplantar injection of ET 5 hr prior to analysis. The glabrous skin of the ipsilateral footpad was collected using a razor blade and minced with scissors in 5 mL of PBS. The tissue was pelleted and resuspended in 2 mL of HEPES-buffered saline (Sigma) containing collagenase A (1 mg/kg, Roche Applied Sciences) and dispase II (2.4 U/mL, Roche Applied Sciences) for 2 hr at 37°C. After incubation, cells were dissociated using a 16-gauge needle attached to a 10 mL syringe, filtered through a 70 µm strainer, and washed with 20 mL of HBSS (Thermo Fisher Scientific) with 0.5% BSA (Sigma). Cells were resuspended in 500 µL of washing buffer and incubated on ice with mouse FcR Blocking Reagent (Miltenyi Biotec) for 10 min, then for 30 min on ice with the following antibodies: anti-CD45-APC/Cy7 (1:200, Biolegend), anti-Ly6G-A488 (1:200, Biolegend), anti-CD11b-BV605 (1:200, Biolegend), and Fixable Viability Dye eFluor-506 (1:1,200, Thermo Fisher). Cells were centrifuged for 5 min at 300 xg and resuspended in 500 µL of washing buffer with 2% PFA. Flow cytometry was performed on a LSR II flow cytometer (BD Biosciences) using BD FACSDiva software (BD Biosciences). Data was analyzed and plotted using FlowJo software (FlowJo LLC).

### Cytokine Analysis

Mice were given intraplantar injection of ET 5 hr prior to analysis. The glabrous skin of the ipsilateral footpad was collected using a razor blade and minced with scissors in 500 uL of PBS. The tissue was further homogenized in a TissueLyser II (Qiagen) for 10 min at 30 Hz, and centrifuged at 18,000 xg for 10 min at 4°C. The supernatant was stored at -80°C until multiplex analysis of cytokine levels, performed by Eve Technologies.

### Statistical Analysis

Statistical tests and significance levels are reported in figure legends. Data are represented as mean ± standard error (SEM). All statistical analyses were performed using GraphPad Prism 8 and 9. All t-tests were performed as two-tailed.

## Results

### Subcutaneous Footpad Infection of *B. anthracis* Sterne Produces Mortality, Edema, and Body Weight Gain

To determine whether nociceptive neurons play a role during *B. anthracis* infection, we utilized a subcutaneous infection model in the footpad of C57BL/6 mice. This model was previously reported to induce robust edema and host immune response at the site of infection (14). We used the attenuated Sterne strain, which lacks the virulent capsule of *B. anthracis* but expresses all components of anthrax toxin, and is widely used for laboratory research (16). Vegetative bacteria were used to take advantage of active toxin production.

**Figure 1:**
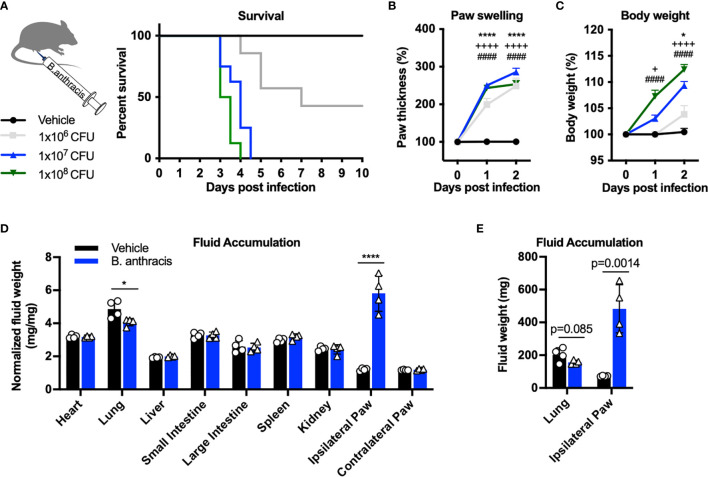
Subcutaneous footpad infection of *Bacillus anthracis* induces significant paw swelling and body weight gain. **(A–C)** C57BL/6 mice received subcutaneous footpad injection of Vehicle (PBS) (n=7) or 1×10^6^, 1×10^7^ or 1×10^8^ CFUs of *(B) anthracis* Sterne (n=7-8). Survival, thickness of the ipsilateral paw and body weight were measured daily. **(D, E)** Fluid weight in internal organs, normalized to the respective dry weights **(D)** or in absolute values **(E)**, following subcutaneous infection with Vehicle (PBS) (n=4) or 1×10^7^ CFU of B. anthracis Sterne (n=4). Statistical analysis: **(A, F)** Log-rank (Mantel-Cox) test, ****p < 0.0001. **(B, C)** Two-way ANOVA with Dunnett’s post-test. Vehicle *vs.* 1×10^6^ CFUs, ^+^p < 0.05, *p < 0.05, ****p < 0.0001. Vehicle *vs.* 1×10^7^ CFUs, ^++++^p < 0.0001. Vehicle *vs.* 1×10^8^ CFUs, ^####^p < 0.0001. **(D, E)** Two-way ANOVA with Sidak’s post-test. *p < 0.05, ****p < 0.0001.

Subcutaneous *B. anthracis* infection caused a severe phenotype including mortality, with higher doses of bacteria inducing greater lethality (**Figure 1A**). Infection was accompanied by significant local swelling in the footpad and rapid increase in body weight during the first 2 days post-infection (**Figures 1B, C** **)**. Given the severe edema observed in the footpad and adjacent hind limb, we hypothesized that the body weight gain was driven by accumulation of fluid. We thus measured the dry *vs.* wet weight of tissues post-infection to determine fluid weights. While we did not observe significant changes in the fluid content of most internal organs following infection (**Figure 1D**), the ipsilateral footpad gained fluid that accounted for approximately 400 mg in weight at the day 2 time point (**Figure 1E**). We observed a slight decrease in fluid content in the lungs, consistent with a previous study which performed systemic injection of ET (17). These data indicated major tissue edema and fluid accumulation occurring in the site of infection in the first 48 hours.

### *B. anthracis*-Induced Mortality, Tissue Swelling and Allodynia Is Mediated by Edema Factor

**Figure 2:**
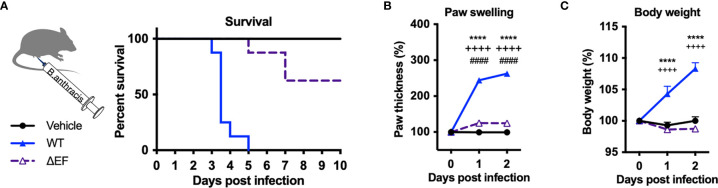
Edema factor mediates *B. anthracis*-induced lethality, paw swelling and body weight gain. **(A–C)** C57BL/6 mice received subcutaneous footpad injection of Vehicle (PBS) (n=8), wild-type *B. anthracis* Sterne (1×10^7^ CFUs; n=8), or an isogenic mutant lacking EF (ΔEF, 1×10^7^ CFUs; n=8). Survival, thickness of the ipsilateral paw and body weight were measured daily. Statistical analysis: **(A)** Log-rank (Mantel-Cox) test, ****p < 0.0001. **(B, C)** Two-way ANOVA with Tukey’s post-test. Vehicle *vs.* WT, ****p < 0.0001; WT *vs.* ∆EF, ^++++^p < 0.0001; Vehicle *vs.* ∆EF, ^####^p < 0.0001.

Edema Toxin (ET), which consists of Protective Antigen (PA) and Edema Factor (EF), is a major virulence factor of *B. anthracis* and known to cause severe swelling, edema, tissue damage and death in experimental animals (9, 10). To determine whether ET is responsible for pathogenesis and localized swelling during *B. anthracis* infection, we performed subcutaneous infection with an isogenic mutant *B. anthracis* strain lacking EF (ΔEF). Deficiency in EF led to significantly increased survival following infection compared to WT *B. anthracis* (**Figure 2A**). Furthermore, ΔEF mutant bacteria caused significantly less paw swelling in the footpad and body weight accumulation in mice compared to WT bacteria (**Figures 2B, C** **)**. These results show that EF is a major virulence factor that mediates mortality, edema, and weight gain during *B. anthracis* infection.

**Figure 3:**
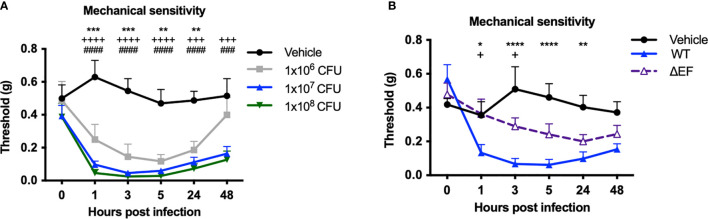
Edema Factor contributes to mechanical sensitivity induced by *Bacillus anthracis* infection. **(A)** C57BL/6 mice received subcutaneous footpad injection of Vehicle (PBS) (n=7) or 1×10^6^, 1×10^7^ or 1×10^8^ CFUs of *B. anthracis* Sterne (n=7-8). **(B)** C57BL/6 mice received subcutaneous footpad injection of Vehicle (PBS) (n=8), wild-type *B. anthracis* Sterne (1×10^7^ CFUs; n=8), or an isogenic mutant lacking EF (ΔEF, 1×10^7^ CFUs; n=8). Mechanical sensitivity of the ipsilateral footpad was measured using von Frey filaments. Statistical analysis: **(A)** Two-way ANOVA with Dunnett’s post-test. Vehicle *vs.* 1×10^6^ CFUs, **p < 0.01, ***p < 0.001. Vehicle *vs.* 1×10^7^ CFUs, ^+++^p < 0.0001, ^++++^p < 0.0001. Vehicle *vs.* 1×10^8^ CFUs, ^###^p < 0.0001, ^####^p < 0.0001. **(B)** Two-way ANOVA with Tukey’s post-test. Vehicle *vs.* WT, *p < 0.05, **p < 0.01, ***p < 0.001,****p < 0.0001. WT *vs.* ∆EF, ^+^p < 0.05.

We next measured whether infection induced changes in mechanical pain hypersensitivity and allodynia (sensitivity to normally innocuous stimuli) using von Frey filaments. Subcutaneous infection in mice induced mechanical allodynia over the first 48 hours of infection, prior to the lethal time points (**Figure 3A** **)**. Mechanical allodynia was significantly reduced in ΔEF *B. anthracis* infected mice compared to WT bacteria-infected mice (**Figure 3B**), indicating that EF contributes to the pain-like behaviors during infection. The partial attenuation of pain suggested that additional factors were induced which work to sensitize nociceptive neurons, such as other damage-associated signals or inflammatory mediators.

### Edema Toxin Is Sufficient to Induce Tissue Swelling, Vascular Leakage, Neutrophil Recruitment and Inflammatory Cytokine Production

**Figure 4:**
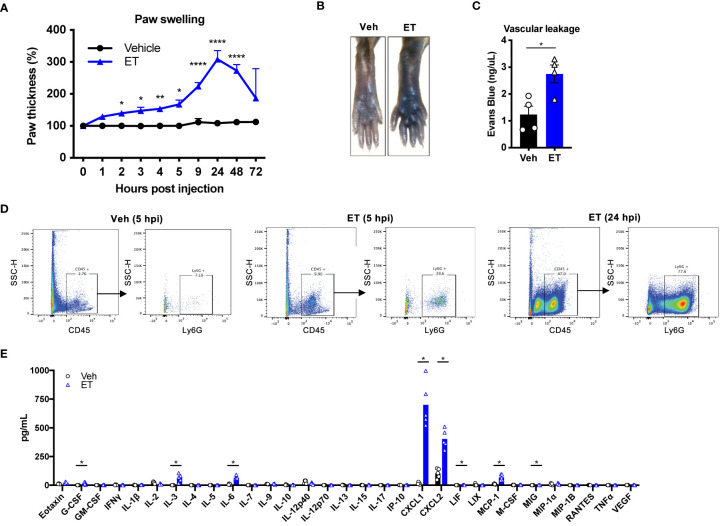
Edema toxin drives significant edema and inflammatory cytokine production. **(A)** Mice received subcutaneous footpad injection of Vehicle (PBS) (n=6) or ET (2 µg PA + 2 µg EF) (n=6). Paw thicknesses were measured at the indicated time points. **(B, C)** Mice received retro-orbital injection of Evans Blue (25 mg/kg) 30 min prior to subcutaneous footpad injection of Vehicle (PBS) (n=4) or ET (2 µg PA + 2 µg EF) (n=4). **(B)** Representative images of ipsilateral footpads at 5 hpi. **(C)** The concentration of Evans Blue dye in clarified tissue lysate of the footpad at 5 hpi. **(D)** Mice received subcutaneous footpad injection of Vehicle (PBS) or ET (2 µg PA + 2 µg EF). Infiltration of CD45^+^Ly6G^+^ neutrophils in the footpad was measured at 5 or 24 hpi by flow cytometry. Data is from tissue combined from 3 different animals. **(E)** Mice received subcutaneous footpad injection of Vehicle (PBS) or ET (2 µg PA + 2 µg EF). Multiplex cytokine analysis was performed in clarified lysate of the footpad harvested at 5 hpi (n=5). Statistical analysis: **(A)** Two-way ANOVA with Sidak’s post-test. *p < 0.05, **p < 0.01, ****p < 0.0001. **(C)** Unpaired t-test. *p < 0.05. **(E)** Mann-Whitney test with the two-stage linear step-up procedure of Benjamini, Krieger, and Yekutieli, 5% FDR. Discoveries are marked by asterisks.

Given the contribution of ET to tissue swelling during *B. anthracis* infection, we wished to interrogate its direct and independent effects on edema and localized inflammatory processes. We thus performed subcutaneous injection of ET into the footpad of mice. Subcutaneous ET produced significant swelling of the paw (**Figure 4A**) as previously reported (17), consistent with our observations during *B. anthracis* infection. We observed two distinct phases of paw swelling: an early phase lasting for several hours post-injection (hpi), where the thickness of the paw increased by 50%, followed by a late phase at 24 hpi, where a 200% increase in paw thickness was observed. In contrast to bacterial infection, we did not observe changes in body weight, suggesting that the effects of ET injection are locally confined. We also found that ET induces vascular effusion at the injection site, measured by extravasation of Evans Blue dye at 5 hpi (**Figures 4B, C** **)**. This observation was consistent with previous reports (11, 12).

We next wished to determine the cellular components of ET-induced swelling. ET is known to inhibit host innate and adaptive immune responses in concert with LT (18), and has been observed to induce neutrophil infiltration in rabbits (12). We found that subcutaneous injection of ET induces significant influx of CD45^+^Ly6G^+^ neutrophils to the footpad of mice at 5 hpi, which continued to increase by 24 hpi (**Figure 4D**). To determine potential molecular mediators of this effect, we screened for cytokine expression in the footpad at 5 hpi. We found that ET injection induces high levels of CXCL1 and CXCL2 (**Figure 4E**), consistent with influx of neutrophils. We also observed that ET induces significant levels of IL-3, IL-6, MCP-1, G-CSF, LIF and MIG compared to vehicle injection, but to smaller magnitudes. Altogether, our results indicated that ET is sufficient to elicit a potent host immune response with both vascular and cellular components.

### Nociceptive Neurons Mediate Edema and Vascular Leakage Induced by Edema Toxin

**Figure 5:**
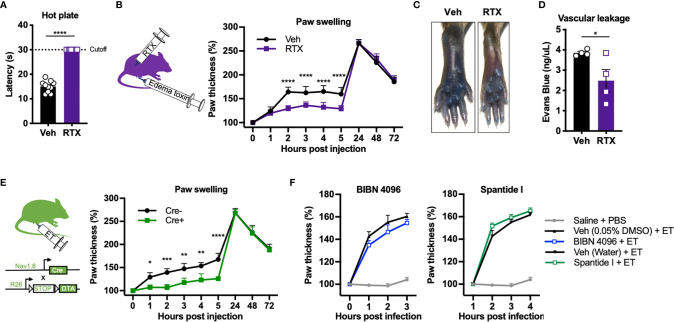
Nociceptive neurons mediate acute edema and vascular leakage caused by Edema toxin injection. **(A)** Responses to the hot plate test was measured in mice treated with RTX or Vehicle (n=10). **(B)** Mice treated with RTX or Vehicle received subcutaneous footpad injection of ET (2 µg PA + 2 µg EF) (n=8-9). Paw thicknesses were measured at the indicated timepoints. **(C, D)** RTX- or Vehicle-treated mice received retro-orbital injection of Evans Blue (25 mg/kg) 30 min prior to subcutaneous footpad injection of ET (2 µg PA + 2 µg EF) (n=4). **(C)** Representative images of ipsilateral footpads at 5 hpi. **(D)** The concentration of Evans Blue dye in clarified tissue lysate of the footpad at 5 hpi. **(E)** Na_v_1.8^cre/+^/DTA (Cre+) or Na_v_1.8^+/+^/DTA littermate controls (Cre-) received subcutaneous footpad injection of ET (2 µg PA + 2 µg EF) (n=3-4). Paw thicknesses were measured at the indicated timepoints. **(F)** Wild-type mice received intraplantar injection of Saline, BIBN 4096 (50 pmol), the vehicle for BIBN 4096 (0.05% DMSO), Spantide I (5 nmol), or the vehicle for Spantide I (water), 15 min prior to PBS or ET (2 µg PA + 2 µg EF). The Saline + PBS group is identical in the two panels. No significant differences were detected between the respective antagonist and vehicle treatment. Statistical analysis: **(A, D)** Unpaired t-test. *p < 0.05. **(B, E)** Two-way ANOVA with Sidak’s post-test. *p < 0.05, **p < 0.01, ***p < 0.001, ****p < 0.0001.

We next wished to determine whether nociceptive neurons contribute to ET-induced swelling and vascular leakage in the footpad. To this end, we chemically ablated nociceptive neurons using Resiniferatoxin (RTX), a potent analog of capsaicin which induces calcium overload and death of Trp_v_1^+^ nociceptive neurons (19). Trp_v_1 is the major ion channel responsible for detecting heat. RTX-treated mice showed significantly attenuated responses on a hot plate test (**Figure 5A**), indicating ablation of Trp_v_1^+^ nociceptive neurons. RTX and vehicle-treated mice were rested for 4 weeks before injection with ET. Nociceptor ablation by RTX significantly attenuated paw swelling hours after ET injection, but not at later time points (**Figure 5B**). RTX treatment also significantly reduced ET-induced vascular effusion of Evans Blue dye at 5 hpi (**Figures 5C, D** **)**.

To further confirm the contribution of nociceptive neurons, we used an orthogonal, genetic method to ablate nociceptors by conditionally expressing the A chain of Diphtheria toxin (DTA) in Na_v_1.8^+^ neurons. DTA inhibits protein translation in mammalian cells through inactivation of eukaryotic elongation factor 2 (eEF-2), causing cell death. We bred Na_v_1.8^cre/+^ mice with ROSA-DTA mice containing a floxed-STOP cassette, selectively killing Na_v_1.8^+^ nociceptive neurons during development (herein referred to as Na_v_1.8^cre/+^/DTA mice). Genetic ablation of Na_v_1.8^+^ nociceptive neurons significantly attenuated ET-induced paw swelling during the early phase but not the late phase (**Figure 5E**), phenocopying our results with RTX-treated animals. Trp_v_1 and Na_v_1.8 mark distinct but overlapping subsets of nociceptive sensory neurons.

### ET-Induced Edema Is Independent of Neuronal ANTXR2, CGRP Signaling and Substance P Signaling

Previously, we found that nociceptive sensory neurons express ANTXR2 and are susceptible to intoxication by ET (8). We thus wished to determine whether direct targeting of nociceptors by ET through ANTXR2 played a role in mediating edema and swelling. To this end, we utilized conditional ANTXR2 KO mice (Na_v_1.8^cre/+^/Antxr2^fl/fl^) lacking receptor function from Nav1.8^+^ nociceptive neurons. We did not observe a difference in ET-induced paw swelling between Na_v_1.8^cre/+^/Antxr2^fl/fl^ mice and their control Na_v_1.8^+/+^/Antxr2^fl/fl^ littermates at any time point (**Supplementary Figure 1**), suggesting that ET induces edema independent of targeting neuronal ANTXR2.

The neuropeptides Calcitonin gene-related peptide (CGRP) and Substance P (SP) are major mediators of neurogenic inflammation. To determine whether CGRP or SP are involved in ET-induced edema, we utilized BIBN 4096, a small molecule antagonist of the CGRP receptor RAMP1, and Spantide I, a peptide antagonist of the SP receptor NK1R. Intraplantar administration of BIBN 4096 or Spantide I 15 min prior to ET did not significantly affect paw swelling (**Figure 5F**), suggesting that alternative neural mediators are involved in regulating the edema.

### Nociceptive Neurons Modulate Neutrophil Influx Induced by Subcutaneous ET Injection

**Figure 6:**
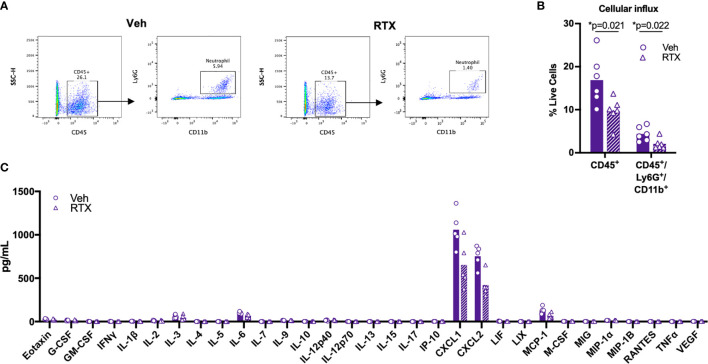
Nociceptors contribute to Edema Toxin-induced neutrophil influx. **(A, B)** RTX- or Vehicle-treated mice received subcutaneous footpad injection of ET (2 µg PA + 2 µg EF). Infiltration of CD45^+^Ly6G^+^CD11b^+^ neutrophils in the footpad was measured at 5 hpi by flow cytometry (n=6). **(A)** Representative dot plots. **(B)** Quantification of CD45^+^ or CD45^+^Ly6G^+^CD11b^+^ cells as a fraction of live cells. **(C)** RTX- or Vehicle-treated mice received subcutaneous footpad injection of ET (2 µg PA + 2 µg EF). Multiplex cytokine analysis was performed in footpad lysate harvested at 5 hpi (n=5). Statistical analysis: **(B)** Unpaired t-test with Holm-Sidak correction. *p < 0.05. **(C)** Mann-Whitney test with the two-stage linear step-up procedure of Benjamini, Krieger, and Yekutieli, 5% FDR. Discoveries are marked by asterisks.

To determine whether nociceptive neurons play a role in the inflammatory response induced by ET, we administered ET into the footpad of nociceptor-ablated mice. We found that RTX treatment significantly attenuated infiltration of CD45^+^ leukocytes and CD45^+^Ly6G^+^CD11b^+^ neutrophils into the footpad at 5 hpi (**Figures 6A, B** **)**. Levels of CXCL1 and CXCL2 were not significantly attenuated, while trending towards a decrease (**Figure 6C**). Collectively, our results suggested that nociceptive neurons regulate processes downstream of cytokine expression to impact vascular permeability, edema and cellular influx in response to ET.

### Nociceptor Ablation Attenuates the Early Phase of Paw Swelling and Overall Body Weight Gain Induced by *B. anthracis* Infection

**Figure 7:**
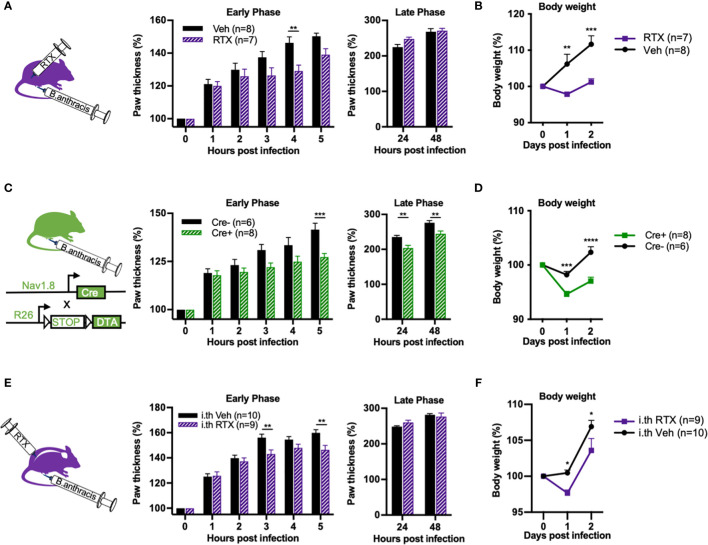
Nociceptor ablation attenuates *Bacillus anthracis*-induced body weight gain and early phase of paw swelling. **(A, B)** Mice treated with subcutaneously administered RTX or Vehicle received subcutaneous footpad injection of 1×10^7^ CFUs of *B. anthracis* Sterne. Thickness of the ipsilateral paw and body weight were measured at the indicated time points. **(C, D)** Na_v_1.8^cre/+^/DTA (Cre+) or Na_v_1.8^+/+^/DTA littermate controls (Cre-) received subcutaneous footpad injection of 1×10^7^ CFUs of *B. anthracis* Sterne. Thickness of the ipsilateral paw and body weight were measured at the indicated time points. **(E, F)** Mice treated with intrathecally administered RTX or Vehicle received subcutaneous footpad injection of 1×10^7^ CFUs of *B. anthracis* Sterne. Thickness of the ipsilateral paw and body weight were measured at the indicated time points. Statistical analysis: **(A–F)** Two-way ANOVA with Sidak’s post-test. *p < 0.05, **p < 0.01, ***p < 0.001, ****p < 0.0001.

We wished to confirm whether nociceptive neurons modulate edema in a physiologically relevant context during *B. anthracis* infection. To this end, we performed subcutaneous footpad infection of *B. anthracis* in mice that underwent chemical ablation of nociceptors *via* systemic injection of RTX or genetic ablation using Na_v_1.8^cre/+^/DTA mice. Consistent with our observation with ET injection, paw swelling induced by *B. anthracis* was attenuated during the early hours of injection in nociceptor ablated mice (**Figures 7A, C** **)**. *B. anthracis*-induced body weight gain was also significantly attenuated (**Figures 7B, D** **)** and notably beyond the early hours of infection. This observation suggested that nociceptive neurons may play a broader role in regulating body weight beyond regulating local edema during early timeframes. Nociceptor ablation did not significantly affect survival post infection (**Supplementary Figures 2A, B** **)**. Nociceptor ablation also did not significantly affect bacterial load in the footpad or dissemination to the popliteal lymph node, liver or spleen at 5 and 48 hpi, time points at which differences in local edema in the footpad and body weight were visible, respectively (**Supplementary Figures 3A, B** **)**.

Next, we sought to investigate whether Trpv1^+^ dorsal root ganglia (DRG) neurons or vagal neurons specifically mediated the observed phenotype in regulating *B. anthracis*-induced edema. While DRG neurons mediate skin neurogenic inflammation, vagal ganglia neurons may also influence body fluid accumulation and sympathetic tone (20). To this end, we performed intrathecal injections of RTX to specifically ablate Trpv1^+^ nociceptive neurons in the DRG without affecting vagal Trpv1^+^ neurons (6). Treatment with intrathecal RTX significantly attenuated the footpad response to noxious heat (**Supplementary Figure 4**), confirming successful ablation. Paw swelling during the early hours of footpad infection with *B. anthracis* was significantly attenuated in mice treated with intrathecal RTX compared to vehicle (**Figure 7E**). We also observed attenuation of body weight gain (**Figure 7F**), albeit to a lesser degree compared with systemic administration of RTX. Thus, while we cannot completely rule out potential contributions from vagal sensory neurons, our results indicate that Trpv1^+^ DRG neurons play a significant role in regulating paw swelling and body weight during *B. anthracis* infection.

Altogether, our results demonstrate that nociceptor neurons modulate local tissue swelling during the early phases of *B. anthracis* infection, and also exert potentially longer lasting effects to modulate the infection-induced gain in body weight.

## Discussion

Beyond their role in detecting danger and signaling pain, nociceptive neurons can modulate host immune responses to bacterial infection. Here, we find that nociceptor neurons contribute to driving the immediate vascular effusion and neutrophil influx induced by ET, promoting rapid and local swelling of tissue within hours. We also found that subcutaneous infection of *B. anthracis* in mice produces mechanical allodynia, partially mediated by ET.

*B. anthracis* is the causative agent of anthrax. Although anthrax primarily afflicts domestic and wild herbivores, it remains a public health concern as an endemic disease in parts of the developing world and as a potential weapon of biological warfare. However, the pathogenic mechanisms by which *B. anthracis* impacts host physiology and produces major phenotypes such as edema are still being elucidated. The nervous system and neurogenic inflammation have not been previously linked to anthrax pathogenesis. One interesting observation we made was that *B. anthracis* infection led to a rapid increase in body weight, and that this phenotype was dependent on nociceptive neurons. It is interesting to note that in many other types of bacterial infections, body weight decreases rather than increases due to sickness behaviors. While the local edema and fluid accumulation in the footpad contributed to this increase in body weight, it did not completely account for the body weight gain during infection. It is possible that nociceptive neurons innervating other organ systems could be controlling both fluid accumulation and immune cell influx in a dispersed manner.

While Edema Toxin has been long known to contribute to local tissue swelling and edema, the exact mechanisms leading to the vascular and immune changes related to this phenotype is still not completely known. We observed that ET induces significant tissue swelling, vascular leakage and cytokine production. Neurons in particular contributed to the early phase of tissue swelling and vascular leakage as well as the recruitment of neutrophils. The cellular and molecular mechanisms of how nociceptive neurons regulate ET-induced edema and immune cell influx remains to be determined. We found that blockade of CGRP signaling *via* RAMP1 using BIBN 4096, or blockade of SP signaling *via* NK1 receptors using Spantide I did not affect ET-induced swelling. However, pharmacological blockade (rather than genetic ablation) may be insufficient, or alternative receptors may be involved. Recently, SP has been found to act on MRGPRB2 in mast cells to induce neurogenic inflammation (21), and this pathway may be pursued in future studies. Vascular smooth muscle cells and vascular endothelial cells are also known targets of ET given their expression of ANTXR2 (17). Neurons may signal directly or indirectly to these cells, concurrent with ET-induced signaling, to modulate vascular inflammation. Neurons could also act on these cells to induce expression of chemokines, adhesion molecules or other factors important for recruitment of neutrophils (22).

The signals that drive nociceptive activation during *B. anthracis* infection remain to be determined. The modeling of subcutaneous footpad infection in mice showed that mechanical allodynia was induced, which could relate to the major tissue swelling that occurs locally due to the edema, or to other damage-associated signals released at the site of infection. This indicates potential pain hypersensitivity, which is in contrast to clinical reports that cutaneous anthrax in humans produce characteristically painless lesions (23, 24). This difference in pain phenotype may be due to disparate sites of infection, as our infection model was established using *B. anthracis* subcutaneous injections in mice, unlike cutaneous anthrax infections which occur through barrier-disrupted skin in humans. Different sites of infection also produce divergent pain symptoms in human, as gastrointestinal anthrax produces ulcerative lesions and abdominal pain (25). Here, we found that subcutaneous injection of ET induces expression of various cytokines including IL6, CXCL1, and CXCL2. CXCL1 in particular can directly activate DRG sensory neurons and produce hyperalgesia (26), and has been shown to contribute to the development of hyperalgesia in a model of inflammatory pain (27). ET may induce expression of these cytokines during *B. anthracis* infection in mice, which may contribute to the development of mechanical hypersensitivity. The mechanisms of pain and role of nociceptors during *B. anthracis* infection in humans remain to be further investigated.

In summary, we report a novel role for nociceptive neurons in the early phase of local edema and cellular influx induced by ET injection and *B. anthracis* infection. Therefore, neurogenic inflammation could play a key part of pathology and inflammation during infection. However, many open questions remain. The overall role of early and later stage edema in driving *B. anthracis* infection remains to be defined, despite it being a common feature of anthrax and the known importance of ET as a critical virulence factor. We did not find evidence that the edema regulated by nociceptors significantly influences bacterial dissemination or infection-induced mortality. Alternatively, *B. anthracis* may be taking advantage of the increase in nutrient availability that results from the increasing leakiness of the vasculature, which is partially mediated through nociceptive neurons. Altogether, our findings highlight nociceptors as a key modulator of early inflammation and edema induced by ET, and suggest a complex interplay between nociceptive neurons, immune cells and bacterial toxins in anthrax pathogenesis.

## Data Availability Statement

The raw data supporting the conclusions of this article will be made available by the authors, without undue reservation.

## Ethics Statement

The animal study was reviewed and approved by Institutional Animal Care and Use Committee (IACUC) at Harvard Medical School.

## Author Contributions 

NY and IC conceived the project. NY, DN, LD, MH, AK-C, and VT performed experiments and data analysis. JP advised experiments and provided *B. anthracis* strains. NY and IC wrote the manuscript with input from all authors. All authors contributed to the article and approved the submitted version.

## Funding

This study was supported by the National Institutes of Health (NIH) (DP2AT009499, R01AI130019), the Burroughs Wellcome Fund to IC, and an NIH T32 training grant (5T32AG000222) to NY.

## Conflict of Interest

The authors declare that the research was conducted in the absence of any commercial or financial relationships that could be construed as a potential conflict of interest.

## Publisher’s Note

All claims expressed in this article are solely those of the authors and do not necessarily represent those of their affiliated organizations, or those of the publisher, the editors and the reviewers. Any product that may be evaluated in this article, or claim that may be made by its manufacturer, is not guaranteed or endorsed by the publisher.
