# Supplementary material for: Nociceptive Sensory Neurons Mediate Inflammation Induced by Bacillus Anthracis Edema Toxin
Source: Front Immunol. 2021 Aug 3;12:642373. doi: 10.3389/fimmu.2021.642373 (PMC8369925; doi:10.3389/fimmu.2021.642373)
Supplement: Supplementary file 1 [file DataSheet_1.pdf]

Supplementary Fig 1. ET-induced paw swelling is independent of targeting ANTXR2 on nociceptive neurons.

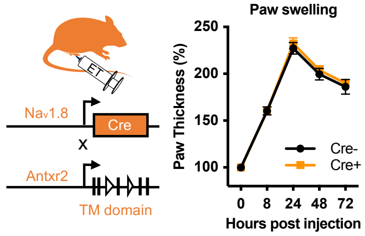

$Na_v1.8^{cre/+}/ANTXR2^{fl/fl}$ (Cre+) conditional KO mice or $Na_v1.8^{+/+}/ANTXR2^{fl/fl}$ littermate controls (Cre-) received subcutaneous footpad injection of ET (2 ug PA + 2 ug EF) (n=5-7). No significant differences were detected.

Supplementary Fig 2. Nociceptor ablation does not affect mortality induced by *Bacillus anthracis* infection.

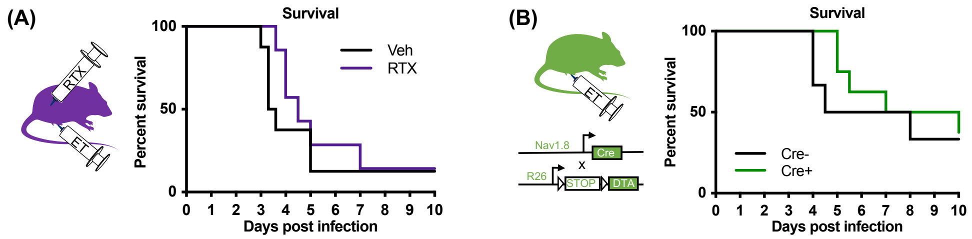

(A) RTX- or Vehicle-treated mice received subcutaneous footpad injection of 1×10^7 CFUs of *B. anthracis* Sterne (n=7-8).

(B) $Na_v1.8^{cre/+}$/DTA (Cre+) or $Na_v1.8^{+/+}$/DTA littermate controls (Cre-) received subcutaneous footpad injection of 1×10^7 CFUs of *B. anthracis* Sterne (n=7-8).

Statistical analysis: (A, B) No significant differences detected by the Log-rank (Mantel-Cox) test.

Supplementary Fig 3. Nociceptor ablation does not affect bacterial load or dissemination following *Bacillus anthracis* infection.

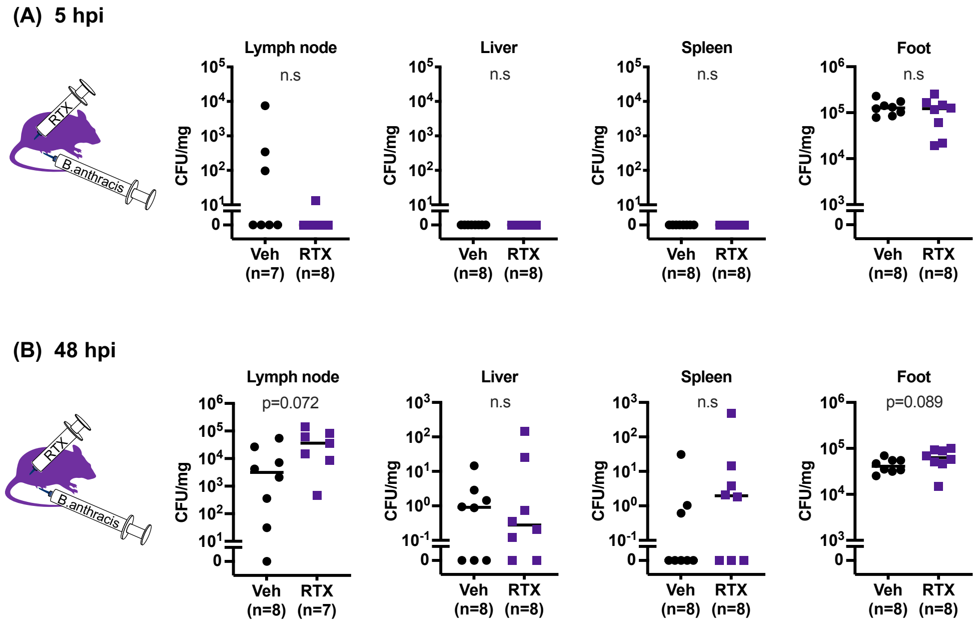

(A-B) RTX- or Vehicle-treated mice received subcutaneous footpad injection of 1×10^7 CFUs of *B. anthracis* Sterne. At **(A)** 5 or **(B)** 48 hours post-infection (hpi), the ipsilateral foot and popliteal lymph node, liver and spleen were harvested. The bacterial load in each organ was normalized to organ weight.

Statistical analysis: (A, B) No significant differences detected by unpaired t-test.

Supplementary Fig 4. Intrathecal administration of RTX significantly attenuates response to noxious heat.

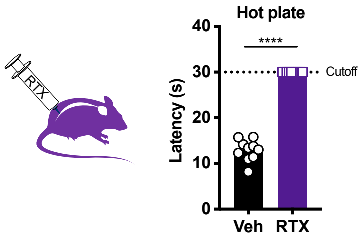

Mice received intrathecal injection of RTX or Vehicle. Following a 4-week rest period, responses to the hot plate test were measured (n=10). **** p<0.0001, unpaired t-test.

Supplementary Fig 5. Protein sequence of EF used in this study.

ANEHYTESDIKRNHKTEKNKTEKEKFKDSINNLVKTEFTNETLDKIQQTQDLLKKIPKDVLEIYSELGGEIYFTDI
DLVEHKELQDLSEEEKNSMNSRGEKVPFASRFVFEKKRETPKLIINIKDYAINSEQSKEVYYEIGKGISLDIISKDK
SLDPEFLNLIKSLSDDSDSSDLLFSQKFKEKLELNNKSIDINFIKENLTEFQHAFSLAFSYYFAPDHRTVLELYAPD
MFEYMNKLEKGGFEKISESLKKEGVEKDRIDVLKGEKALKASGLVPEHADAFKKIARELNTYILFRPVNKLATN
LIKSGVATKGLNVHGKSSDWGPVAGYIPFDQDLSKKHGQQLAVEKGNLENKKSITEHEGEIGKIPLKLDHLRIEE
LKENGIILKGKKEIDNGKKYYLLESNNQVYEFRISDENNEVQYKTKEGKITVLGEKFNWRNIEVMAKNVEGVLK
PLTADYDLFALAPSLTEIKKQIPQKEWDKVVNTPNSLEKQKGVTNLLIKYGIERKPDSTKGTLSNWQKQMLDRL
NEAVKYTGYTGGDVVNHGTEQDNEEFPEKDNEIFIINPEGEFILTKNWEMTGRFIEKNITGKDYLYYFNRSYNKI
APGNKAYIEWTDPITKAKINTIPTSAEFIKNLSSIRRSSNVGVYKDSGDKDEFAKKESVKKIAGYLSDYYNSANHI
FSQEKKRKISIFRGIQAYNEIENVLKSKQIAPEYKNYFQYLKERITNQVQLLLTHQKSNIEFKLLYKQLNFTENETD
NFEVFQKIIDEK
